# Vancomycin‐Mediated Binding of DNA Origami Nanostructures to Gram‐Positive and Gram‐Negative Bacteria

**DOI:** 10.1002/cbic.70436

**Published:** 2026-06-30

**Authors:** Özge Coşkuner Leineweber, Ulrike Hofmann, Guido Grundmeier, Yixin Zhang, Adrian Keller

**Affiliations:** ^1^ Technical and Macromolecular Chemistry Paderborn University Paderborn Germany; ^2^ B CUBE – Center for Molecular Bioengineering Technische Universität Dresden Dresden Germany

**Keywords:** antibiotics, antimicrobial, *Bacillus subtilis*, biosensor, DNA origami, drug delivery, *Escherichia coli*, glycopeptide, vancomycin

## Abstract

DNA origami nanostructures (DONs) have promising applications in biomedicine and biosensing, which often require their efficient binding to target cells. By immobilizing the glycopeptide antibiotic vancomycin on DONs, DON binding to Gram‐positive and Gram‐negative bacteria can be facilitated. Here, we investigate how this multivalent binding is affected by the number and arrangement of the vancomycin modifications on two‐dimensional DONs. We find that for both Gram‐positive *Bacillus subtilis* and Gram‐negative *Escherichia coli*, binding increases with the number of vancomycin modifications per DON. In general, binding to *E. coli* is stronger than to *B. subtilis*, which may be attributed to differences in the architectures of the cell envelopes. Interestingly, for both bacteria, the total number of vancomycin modifications appears to be more important than their arrangement, as DONs with 18 vancomycin molecules on one side show similar binding as DONs with 18 vancomycin molecules distributed over both sides. This enables the attachment of multiple probe molecules to the vancomycin‐free side of the DONs for enhancing detection efficiency without compromising binding affinity. These results may thus provide guidelines for the design and synthesis of vancomycin‐modified DONs for antimicrobial drug delivery and pathogen detection.

## Graphical abstract

Vancomycin‐mediated DNA origami binding to *Bacillus subtilis* and *Escherichia coli* is investigated. Binding to both bacteria increases with the number of vancomycin modifications per DNA origami nanostructure (DON) but is independent of their actual arrangement. This offers the opportunity to attach additional probe molecules to the vancomycin‐free side of a two‐dimensional DON for more efficient pathogen detection.

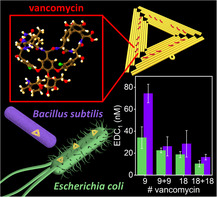

## 1 Introduction

DNA origami nanostructures (DONs) [1, 2] have become an integral part of modern biomedical research [3, 4, 5], serving, for instance, as vehicles for drug [6, 7], enzyme [8, 9], and gene [10, 11] delivery, as diagnostic biosensors [12, 13], and as active pharmaceuticals [14, 15]. In the early days, DON‐related research in biomedicine focused mostly on applications in cancer chemotherapy [16, 17, 18]. However, the last few years have seen growing interest also in other biomedical fields such as anti‐inflammatory therapy [19], immune modulation [20], and infectious diseases [21].

All these applications benefit from the unprecedented molecular control provided by the DNA origami technique, which allows not only for the high‐yield synthesis of well‐defined nanostructures with arbitrary shapes but also for their site‐specific functionalization with nanometer and subnanometer precision [22]. The latter enables the creation of multimeric arrangements of small molecules [8, 15, 23], nucleic acids [8, 24, 25, 26, 27], and proteins [6, 8, 23, 28] on the DON surface. Such modifications may facilitate the specific interaction of the DONs with certain cell types [6, 24, 25] and receptors [6, 23, 27, 28], which can be exploited in the fields of targeted drug delivery [6, 8, 25], cell detection [24, 26, 27], and to modify cell signaling [6, 23, 28]. In all those examples, multivalency plays an important role [29], as multivalent binding of several molecules attached to one DON may result in dramatically enhanced binding affinity. On the other hand, by controlling the spatial arrangement of the molecular binders on the DON surface, target selectivity may be enhanced as well.

In our recent work, we have created multimeric arrangements of the glycopeptide antibiotic vancomycin on two‐dimensional DON triangles [30]. Vancomycin binds to the peptidoglycan precursor peptides in the cell wall of Gram‐positive bacteria and thereby blocks enzymatic cell wall crosslinking [31]. While the monovalent vancomycin–peptide complex has a dissociation constant *K*_d_ of about 1 µM, multivalent complexes may show much stronger binding [32]. Indeed, the multimeric vancomycin–DON conjugates were able to efficiently bind to both Gram‐positive *Bacillus subtilis* and Gram‐negative *Escherichia coli* [30]. Nevertheless, even DON conjugates carrying a maximum number of 36 vancomycin modifications did not exert any antimicrobial activity against both bacteria at vancomycin concentrations up to at least 2.91 and 1.66 µM, respectively, indicating that the comparably large DONs bind only to the cell surface but are incapable of penetrating sufficiently deep into the cell wall to cause significant damage [30].

While our previous work established vancomycin‐decorated DONs as promising constructs for antimicrobial drug delivery and pathogen detection, their binding to bacterial cells has not been investigated in depth yet. The current study thus characterizes the binding of vancomycin‐decorated DON triangles with different vancomycin arrangements to Gram‐positive *B. subtilis* and Gram‐negative *E. coli* for different vancomycin arrangements. Although similar attempts have already been made in the past using vancomycin‐modified inorganic nanoparticles, controlling vancomycin density and arrangements on the nanoparticle surface has been rather challenging [33]. In contrast, by conjugating vancomycin directly to selected DON staple strands using strain‐promoted azide–alkyne cycloaddition (SPAAC), we were able to vary the number of vancomycin molecules per DON from 9 to 36. In general, we observe that a larger number of vancomycin modifications per DON leads to stronger binding to both bacteria. However, all vancomycin‐modified DONs show stronger binding to *E. coli* than to *B. subtilis*. Furthermore, we find for both bacteria that the total number of vancomycin modifications is apparently more important than their arrangement on the DON. A DON with 18 vancomycin molecules on one side shows similar binding as a DON with 18 vancomycin molecules distributed over both sides. As we demonstrate, this effect can be exploited to increase the detection efficiency by attaching additional probe molecules to the free side of the DON. Our results thus provide an important basis for the design of vancomycin‐modified DONs with optimized bacteria‐binding properties.

## 2 Results and Discussion

**FIGURE 1:**
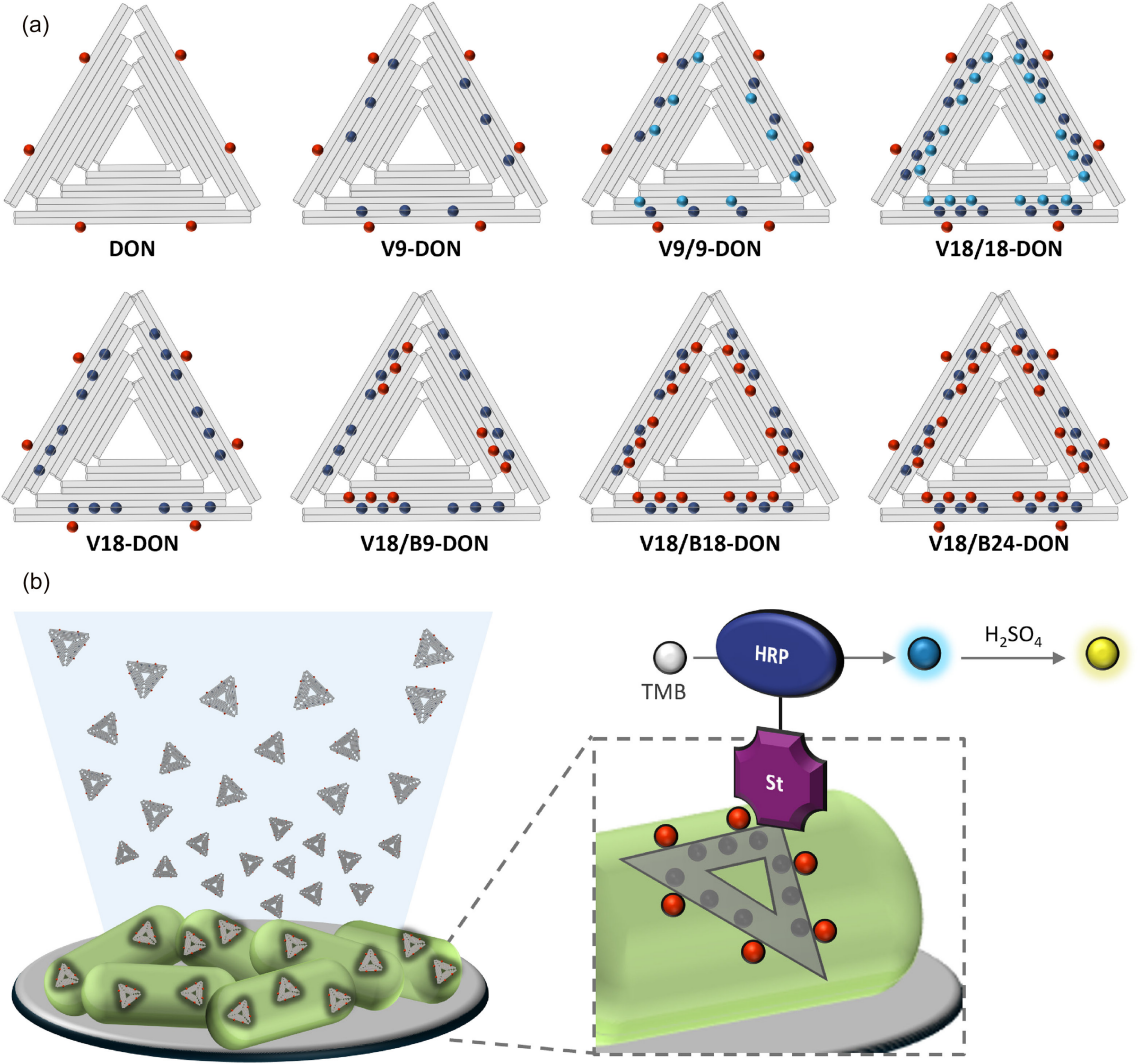
Experimental strategy. (a) Schematic representations of the DON designs investigated in this work. Biotin modifications are indicated in red, vancomycin modifications in blue, with light and dark blue indicating modifications on opposite sides of the DON. (b) Scheme of the ELONA measurements. Vancomycin‐ and biotin‐modified DONs are bound to bacteria adhering to the bottom of a microwell plate. Subsequently, HRP‐conjugated streptavidin (HRP‐St) binds to the biotin modifications and oxidizes TMB into a blue reaction product. Upon addition of H_2_SO_4_, the product is transformed into a stable yellow complex, subsequently used for binding quantification.

Figure 1a shows schematic representations of the different vancomycin‐modified DON triangles investigated in this study. The DONs were modified either on just one side (V9‐DON, V18‐DON) or on both sides (V9/9‐DON, V18/18‐DON), each with either 9 (V9‐DON, V9/9‐DON) or 18 (V18‐DON, V18/18‐DON) vancomycin molecules. The combined conjugation and incorporation yield per vancomycin modification has previously been determined to be 72% on average [30]. Therefore, the distributions of vancomycin modifications per DON were estimated by a computational simulation [30]. For DONs with nominally 9, 18, and 36 vancomycin modifications, the resulting distributions peak at 7, 13, and 26 vancomycin molecules per DON, respectively (see Figure S8). Additionally, the DONs also carry 6–24 biotin modifications. Despite carrying such a large number of staple modifications, we could demonstrate in our previous work using atomic force microscopy and gel electrophoresis that the vancomycin and biotin‐modified DONs fold with high yield and do not show notable aggregation [30]. To some extent, this can be attributed to the peculiarities of the DON triangles selected for these experiments. The Rothemund triangle in general is more rigid than other two‐dimensional DON designs [34], for which the placement of staple modifications on one surface often results in global shape distortions [35]. Furthermore, the DON triangles have only few exposed blunt ends, so that they do not tend to aggregate under physiological conditions [1]. This renders them promising delivery vehicles for various biomedical applications, which has already been demonstrated in several in vivo studies [7, 36, 37, 38, 39, 40].

Binding of the DONs to the bacteria is quantified using an enzyme‐linked oligonucleotide assay (ELONA) [24], in which HRP‐conjugated streptavidin (HRP‐St) binds to the biotin modifications of the cell‐bound DONs (Figure 1b). The immobilized HRP then oxidizes its TMB substrate into a blue reaction product, which is subsequently converted into a stable yellow complex that can be detected via its absorption at 450 nm.

**FIGURE 2:**
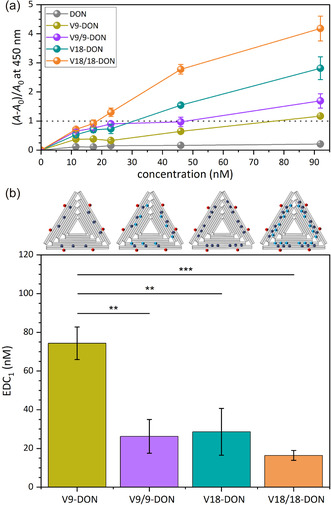
ELONA results for *B. subtilis*. (a) Normalized absorbance at 450 nm obtained for binding of the different DON variants at concentrations ranging from 11.5 nM to 92 nM. (b) Determined effective concentration EDC_1_, i.e., the concentration at which the normalized ELONA signal reaches a value of 1 as indicated by the dotted line in (a). The results are presented as mean ± standard deviation (*n* = 3). Statistical significances were calculated using a two‐sided *t*‐test and are indicated as * (*p* < 0.05), ** (*p* < 0.01), and *** (*p* < 0.001).

First, we investigated the binding of DONs with different vancomycin arrangements to Gram‐positive *B. subtilis*. Figure 2a shows the ELONA results obtained for DON concentrations ranging from 11.5 to 92 nM. While the bare DONs show only negligible binding to the bacteria, binding is notably increased for V9‐DON, which features nine vancomycin modifications on one side. Increasing the number of vancomycin modifications apparently leads to increased binding. To enable a more quantitative evaluation of binding affinity, the effective detection concentration EDC_1_ has been determined. EDC_1_ is the DON concentration at which the normalized ELONA signal (*A *‒ *A*_0_)/*A*_0_  =  1. Here, *A* is the recorded absorbance at 450 nm after DON binding, whereas *A*_0_ is the absorbance of the DON‐free control. This means that at EDC_1_, the baseline absorbance has doubled due to the binding of the DONs to the bacteria. It is thus similar in definition to the well‐established half maximal effective concentration EC50 but does not require the concentration curves to reach saturation. This makes EDC_1_ a more suitable measure for quantitatively assessing differences in DON binding as all vancomycin‐modified DONs reach (*A *‒ *A*_0_)/*A*_0_ = 1 but not necessarily the saturation regime (see Figure 2a). As is shown in Figure 2b, V9‐DON has an EDC_1_ of about 74 nM. Adding the same number of vancomycin molecules also to the other side of the DON (V9/9‐DON) results in a ≈65% reduction in the EDC_1_ to about 26 nM. A similar value is obtained also for V18‐DON, which carries the same number of vancomycin modifications but on one side instead of having them distributed over both sides. Doubling the number of vancomycin molecules per DON by attaching another 18 modifications to the free side (V18/18‐DON) leads to a further reduction in EDC_1_ to about 16 nM. However, the comparatively large error bars obtained for V9/9‐DON and V18‐DON render this small decrease nonsignificant. Nevertheless, these observations suggest that vancomycin‐mediated DON binding to *B. subtilis* scales with the absolute number of vancomycin modifications independent of their actual arrangement on the two DON sides. The origin of this effect probably lies in the competition between initial adsorption geometry and binding strength. Having just one DON side modified halves the probability that it adsorbs with the vancomycin modifications facing the cell wall. However, this reduction in binding probability is then compensated by the stronger binding achieved by doubling the number of vancomycin modifications on the DON side facing the cell surface.

**FIGURE 3:**
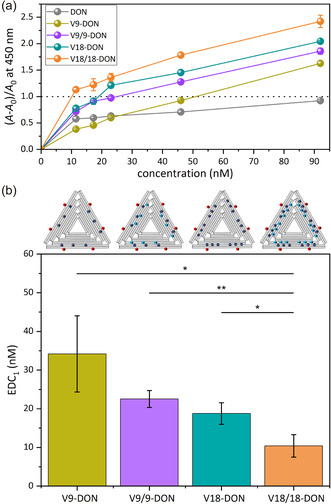
ELONA results for *E. coli*. (a) Normalized absorbance at 450 nm obtained for binding of the different DON variants at concentrations ranging from 11.5 nM to 92 nM. (b) Determined effective concentration EDC_1_, i.e., the concentration at which the normalized ELONA signal reaches a value of 1 as indicated by the dotted line in (a). The results are presented as mean ± standard deviation (*n* = 3). Statistical significances were calculated using a two‐sided *t*‐test and are indicated as * (*p* < 0.05), ** (*p* < 0.01), and *** (*p* < 0.001).

For *E. coli*, the ELONA data in Figure 3a show trends similar to those observed for *B. subtilis* in Figure 2a. However, the determined EDC_1_ values differ notably. Whereas for *B. subtilis*, V9‐DON has an EDC_1_ of 74 nM (Figure 2b), this value drops to about 34 nM for *E. coli* (Figure 3b). Such drops in EDC_1_ are also observed for the other arrangements but are less dramatic, with the observed reduction in EDC_1_ ranging from 20% to 33 %. This stronger binding of the vancomycin‐modified DONs to *E. coli* is rather surprising, considering that monomeric vancomycin is not able to cross the outer membrane of Gram‐negative bacteria. However, DONs [30] and various other nanoparticles [31] have been shown to be able to penetrate the outer membrane and thereby transport the conjugated vancomycin into the cell wall environment. This might explain the increased binding affinity of the vancomycin‐modified DONs to *E. coli* compared to *B. subtilis*. Upon penetrating the outer membrane of *E. coli*, the comparatively large and rigid DONs reach and bind to the dense Gram‐negative cell wall before completely passing through the outer membrane. This partial penetration of the outer membrane then results in a large fraction of the DON sticking out of the membrane, so that its biotin modifications (attached via long T_12_ spacers) are available for HRP‐St binding. At the same time, the DONs become partially embedded in the outer membrane, which might protect them during subsequent washing steps, thus preventing their desorption and increasing overall binding efficiency. Additionally, the different architectures of Gram‐negative and Gram‐positive cell walls might affect binding as well. For instance, the Gram‐positive cell wall of *B. subtilis* contains teichoic acids [41], which are negatively charged and thus lead to electrostatic repulsion between the cell wall and the DONs. Once the DONs have passed through the outer membrane of *E. coli*, however, binding to the Gram‐negative cell wall with its reduced negative charge would be favored. Both effects, i.e., protection during washing and reduced electrostatic repulsion between DONs and Gram‐negative cell wall are in line with the observation that the differences in the binding affinities for *B. subtilis* and *E. coli* become smaller when more vancomycin modifications are added to the DONs. Vancomycin‐mediated binding becomes stronger with increasing numbers of vancomycin modifications, whereas the contributions of those secondary effects remain constant.

The observation that for both bacteria the total number of vancomycin modifications is more important than their arrangement on the DON surface may have important implications for drug delivery and diagnostic applications. In particular, all vancomycin modifications required for efficient binding can be immobilized on one single side of a two‐dimensional DON, so that the vancomycin‐free side may be used to carry other functional molecules or nanoparticles to the bacterial cells. To test the viability of this approach, we next decorated the free DON side with additional biotin modifications.

**FIGURE 4:**
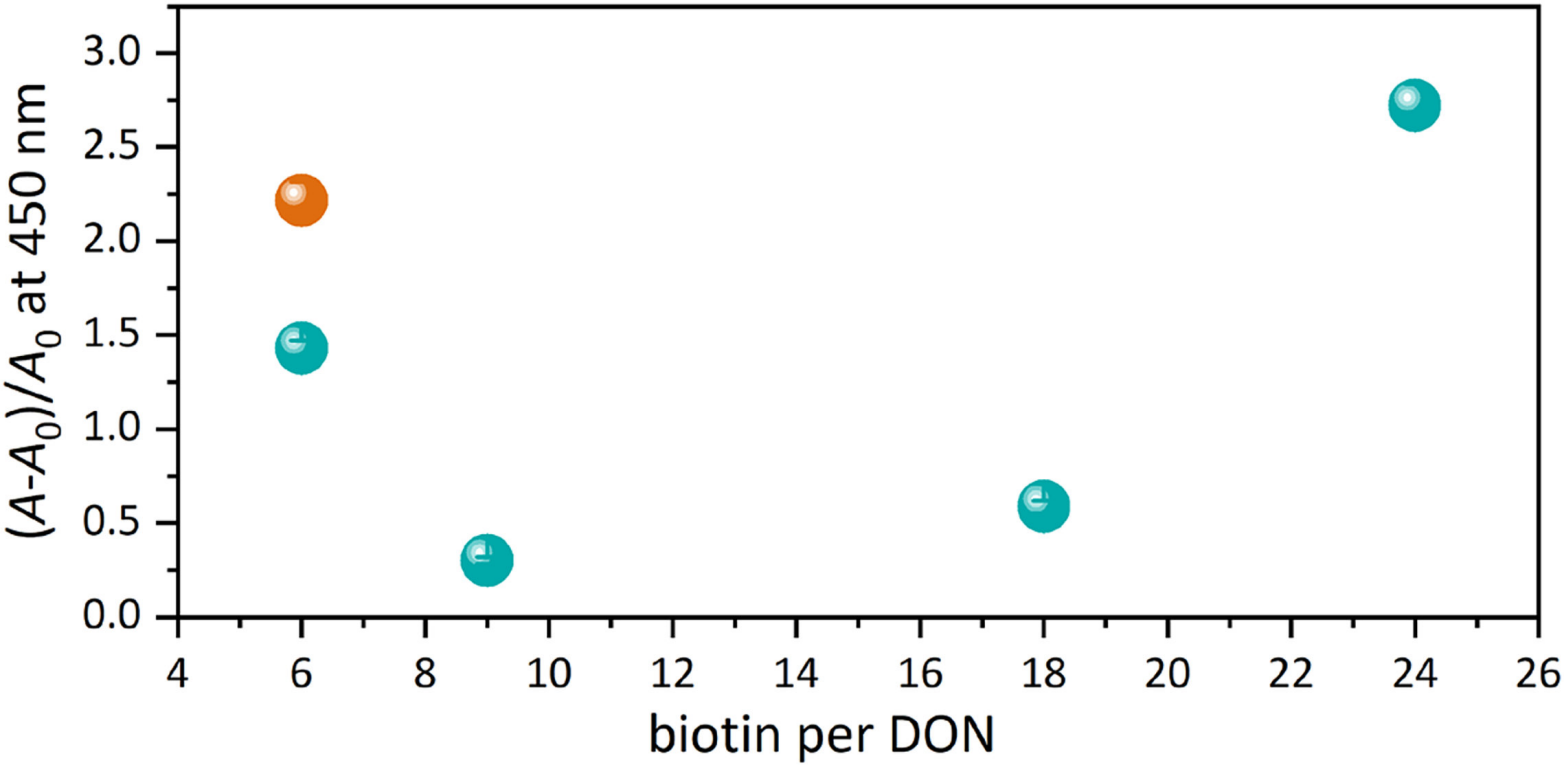
Influence of the number of biotin modifications per DON on the resulting ELONA signal. Normalized absorbance at 450 nm obtained for binding of V18‐DONs (46 nM) with 6–24 biotin modifications (blue data points) to *E. coli*. The orange data point corresponds to V18/18‐DON with six biotin modifications. The results are presented as mean ± standard deviation (*n* = 5). Error bars are smaller than the data points.

In the experiments discussed so far, all DONs featured six biotin modifications, two at each outer edge of the triangle (see Figures 1a and S1–S4). For applications in clinical pathogen detection, it may be beneficial to increase the number of biotin modifications per DON to enhance the ELONA signal and thereby increase the sensitivity of the assay. Therefore, we used V18‐DON with 18 vancomycin modifications on one side and added additional biotin modifications to its other side, creating variants with 9 (V18/B9‐DON), 18 (V18/B18‐DON), and 24 (V18/B24‐DON) biotin modifications (see Figures 1a and S5–S7). Figure 4 shows the normalized ELONA signals obtained for all four variants binding to *E. coli*. Surprisingly, both V18/B9‐DON and V18/B18‐DON produce considerably lower ELONA signals than V18‐DON (featuring six biotins). In these two variants, biotin modifications are located exclusively on inner sites of the DON surface but not on the edges (see Figure 1a), suggesting that edge modifications lead to more efficient recruiting of HRP‐St and/or higher enzyme activity. Four factors could contribute to this effect. First, it is well known, albeit not well understood, that enzyme immobilization on DNA scaffolds affects enzyme activity, which is often increased but sometimes also decreased [42]. Therefore, it is possible that immobilization on inner sites of the DON surface and edge sites results in reduced and increased HRP activity, respectively. Second, the affinity of a protein for DON‐immobilized ligands depends on the exact location of the ligand on the DON due to the presence of different microenvironments along the DON surface [22, 43]. For the DON triangle used in the present study, St‐biotin binding was found to be strongly reduced in the center of trapezoid surfaces [43]. This would result in a lower binding yield of HRP‐St on the DON surface compared to the DON edges and consequently a reduction in ELONA signal. Third, as the biotin modifications are attached to the staple strands via poly‐T spacers, there is a possibility for the single‐stranded biotin‐modified overhangs to thread through the DON and end up on the opposite side [44, 45]. In this case, some of the biotin modifications would be buried between the DON and the bacterial cell surface, thus preventing the binding of HRP‐St and leading to a lower ELONA signal. Fourth, if the DON gets partially embedded in the outer membrane during binding as discussed above, some of the biotin modifications on the DON surface may get buried beneath the membrane, rendering them inaccessible for HRP‐St binding. Due to the large curvature of the *E. coli* cell (0.25–1.0 μm in diameter) [46], however, the edges of the comparably large and rigid DON triangle (about 120 nm edge length) will stick out of the membrane, so that their tethered biotin modifications remain accessible.

Nevertheless, combining both surface and edge modifications in V18/B24‐DON leads to a strong increase in the ELONA signal, which is almost doubled compared to that of V18‐DON (Figure 4). Most intriguingly, the obtained signal is even larger than that of V18/18‐DON (orange data point in Figure 4), which carries 36 vancomycin molecules but only six biotin modifications. This clearly demonstrates that in the ELONA‐based detection of bacteria, a lower number of vancomycin modifications can be compensated by a larger number of biotin modifications, as long as the latter are located at sites that favor strong enzyme binding and activity.

## 3 Conclusions

In summary, we have investigated the role of multivalency in the binding of vancomycin‐modified DONs carrying 9–36 vancomycin molecules to Gram‐positive *B. subtilis* and Gram‐negative *E. coli*. ELONA was used to quantify DON binding, showing that a larger number of vancomycin modifications per DON leads to stronger binding to both bacteria. Most importantly, the binding of all vancomycin‐modified DONs to *E. coli* was found to be notably stronger than for *B. subtilis*, despite *E. coli* being intrinsically vancomycin resistant. This finding might originate in the (partial) penetration of the outer membrane of *E. coli* by the DONs and/or differences in cell wall architecture.

Furthermore, we found that for both bacteria, the absolute number of vancomycin modifications has a stronger influence on DON binding than their actual arrangement on the DON surface. We exploited this fact by decorating the vancomycin‐free side with additional biotin modifications to enhance the ELONA signal. Surprisingly, we observed that the location of the biotin modifications on the DON has a surprisingly strong impact on the obtained ELONA signal, with biotins located on the outer edge of the DON triangle providing much larger signals than biotins immobilized on inner sites of the DON surface. While the origin of these differences is yet to be elucidated, contributing factors may include spatial differences in St binding and/or HRP activity on the DON surface, as well as partial embedding of the DON in the outer membrane of *E. coli*. Nevertheless, by immobilizing 24 biotin molecules on one DON with 18 vancomycin modifications, we obtained a higher ELONA signal than for a DON with 36 vancomycin and six biotin modifications. This may help to increase the assay sensitivity and thus result in a more cost‐efficient diagnostic assay.

For applications of vancomycin‐modified DONs in antimicrobial drug delivery, both low host toxicity and high selectivity of bacterial over host cells are essential. Several previous works have already addressed these issues for other vancomycin–nanoparticle conjugates. In general, toxicity of different vancomycin–nanoparticle conjugates against various mammalian cells was found to be low to negligible [47, 48, 49, 50]. Furthermore, it was demonstrated by Qi et al. that vancomycin‐conjugated silica nanoparticles selectively bind to bacterial cells (*Staphylococcus aureus* and *E. coli*) but not to RAW 264.7 cells [47]. While these observations suggest that vancomycin‐modified DONs may show similar selectivity, future studies should evaluate possible off‐target binding and potential toxicity against mammalian cells.

## 4 Materials and Methods

### 4.1 DNA Origami Folding

Vancomycin and biotin‐modified DON triangles [1] (V/B‐DON) were assembled as previously described [30] by combining M13mp18 scaffold (Bayou Biolabs) with a tenfold molar excess of 208 staple strands (Eurofins Genomics) in 1xTAE buffer (Carl Roth) supplemented with 10 mM MgCl_2_ (Sigma‐Aldrich). Staples modified with vancomycin (Table S1) and biotin (Metabion International, Tables S2 and S3) were incorporated by replacing their unmodified counterparts in the folding mix. 3′‐amino‐modified oligonucleotides (Metabion International) were conjugated to azide‐modified vancomycin via SPAAC, as detailed in our previous work [30]. The DONs were annealed in a thermocycler (T100, Bio‐Rad) by heating the mixture to 80°C, followed by a gradual cooling to room temperature over 90 min. Purification was carried out using two sequential spin filtering steps with 100 kDa MWCO Amicon Ultra centrifugal units (0.5 mL, Merck) at 10,000 rpm for 10 min, including an intermediate wash with PBS buffer supplemented with 10 mM MgCl_2_ to remove residual staples. The concentrations of the purified DONs were quantified by UV/Vis absorption (Nanophotometer P330, Implen).

### 4.2 Bacterial Cell Culture

2.5 g LB broth (Carl‐Roth) and 0.2033 g MgCl_2_ · 6H_2_O (Sigma‐Aldrich) were dissolved in 100 mL molecular biology‐grade water (VWR) and sterilized at 121°C for 15 min. To ensure the structural stability of the DONs, the cultivation medium was supplemented with 10 mM MgCl_2_. For precultures, 500 μL of each bacterial stock (*B. subtilis*, DSM 5545, and *E. coli*, DSM 5695) was inoculated into 40 mL medium and incubated overnight at 37°C in a shaking incubator at 150 rpm. Fresh cultures were then initiated by adding 500 μL of the overnight culture to 40 mL medium and grown under identical conditions until the cells reached exponential growth.

### 4.3 ELONA

The interaction of V/B‐DONs with *B. subtilis* and *E. coli* was evaluated by ELONA [24]. Bacterial cultures were washed twice with PBS and adjusted to OD600 = 0.5 in PBS. 100 µL aliquots of each suspension were added to Nunc‐Immuno MaxiSorp 96‐well microplates (Sigma‐Aldrich) and incubated overnight at 4°C to allow surface immobilization. Wells were then washed thrice with 200 µL of PBS containing 0.05% (v/v) Tween 20 to remove nonadherent cells and minimize nonspecific adsorption. Subsequently, 100 µL of V/B‐DONs or bare DONs, prepared at concentrations ranging from 11.5 to 92 nM in PBS supplemented with 10 mM MgCl_2_, was added to the designated wells and incubated for 1 h at room temperature. Afterwards, the wells were washed thrice with 200 µL of PBS containing 0.02% (v/v) Tween 20 and incubated with 100 µL 125 ng/mL HRP‐St (1.25 mg/mL, Thermo Fisher) for 1 h at room temperature. Excess HRP‐St conjugate was removed by five additional washes with PBS, followed by the addition of 100 µL of TMB substrate solution (1‐Step Ultra TMB‐ELISA Substrate, Thermo Fisher) and subsequent incubation for 15 min. The reaction was stopped by adding 50 µL of 1 M H_2_SO_4_ solution (96%), and the absorbance at 450 nm was measured using a Tristar2 S LB 942 plate reader (Berthold Technologies). Note that two different batches of HRP‐St were used for the *B. subtilis* and the *E. coli* experiments, which produced different absolute ELONA signals. Therefore, absolute absorbance values can be compared only within but not between each series of experiments.

### 4.4 Quantification

From the ELONA data, EDC_1_ values were determined. For this, the recorded absorbance values *A* of each replicated were normalized to those of the DON‐free control *A*_0_, plotted as a function of DON concentration, and fitted using the hyperbola function in Origin 2026. The resulting fit equations were then used to calculate the effective concentration EDC_1_, at which the normalized ELONA signal reached a value of 1. All fits are shown in Figures S10–S17. Non‐normalized absorbance data are shown in Figure S9.

### 4.5 Statistical Analysis

The results are presented as the mean ± standard deviation (*n* = 3 or *n* = 5, see figure captions). Statistical significance was determined by calculating *p* values using a two‐sided *t*‐test (unpaired, homoscedastic) in Microsoft Excel, version 2604.

## Funding

This study was supported by Universität Paderborn.

## Conflicts of Interest

The authors declare no conflicts of interest.

## Supporting information

Supplementary Material

## Data Availability

The data that support the findings of this study are available in the supporting information (SI) of this article.
