# Supplementary material for: Vancomycin‐Mediated Binding of DNA Origami Nanostructures to Gram‐Positive and Gram‐Negative Bacteria
Source: Chembiochem. 2026 Jun 30;27(13):e70436. doi: 10.1002/cbic.70436 (PMC13316729; doi:10.1002/cbic.70436)
Supplement: Supplementary file 1 — Supplementary Material [file CBIC-27-e70436-s001.pdf]

Supporting Information

Vancomycin-Mediated Binding of DNA Origami Nanostructures to Gram-Positive and Gram-Negative Bacteria

Özge Coşkuner Leineweber, Ulrike Hofmann, Guido Grundmeier, Yixin Zhang and Adrian Keller

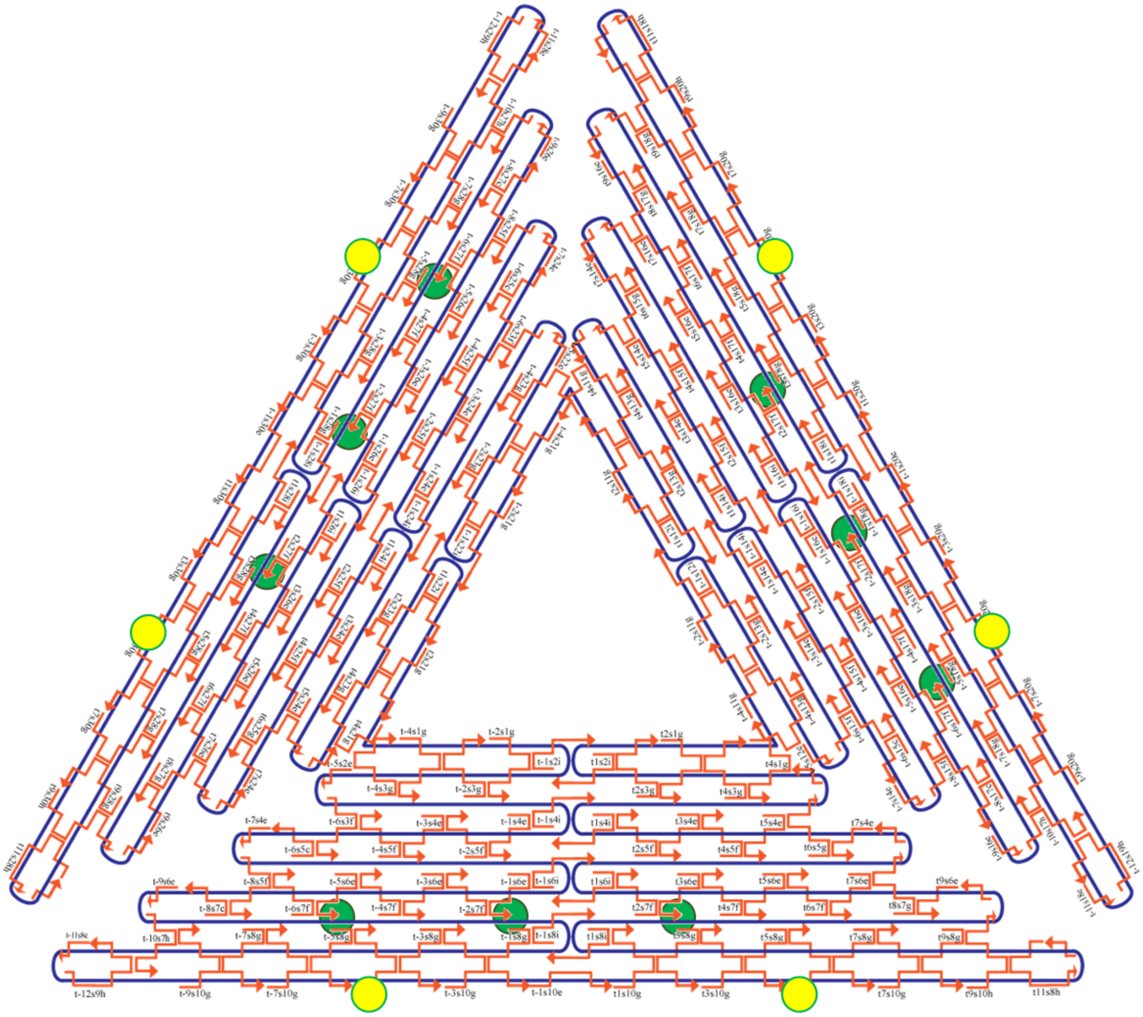

Figure S1. Scaffold routing and staple pattern of V9-DON. Vancomycin sites are indicated in green. Biotin sites are indicated in yellow.

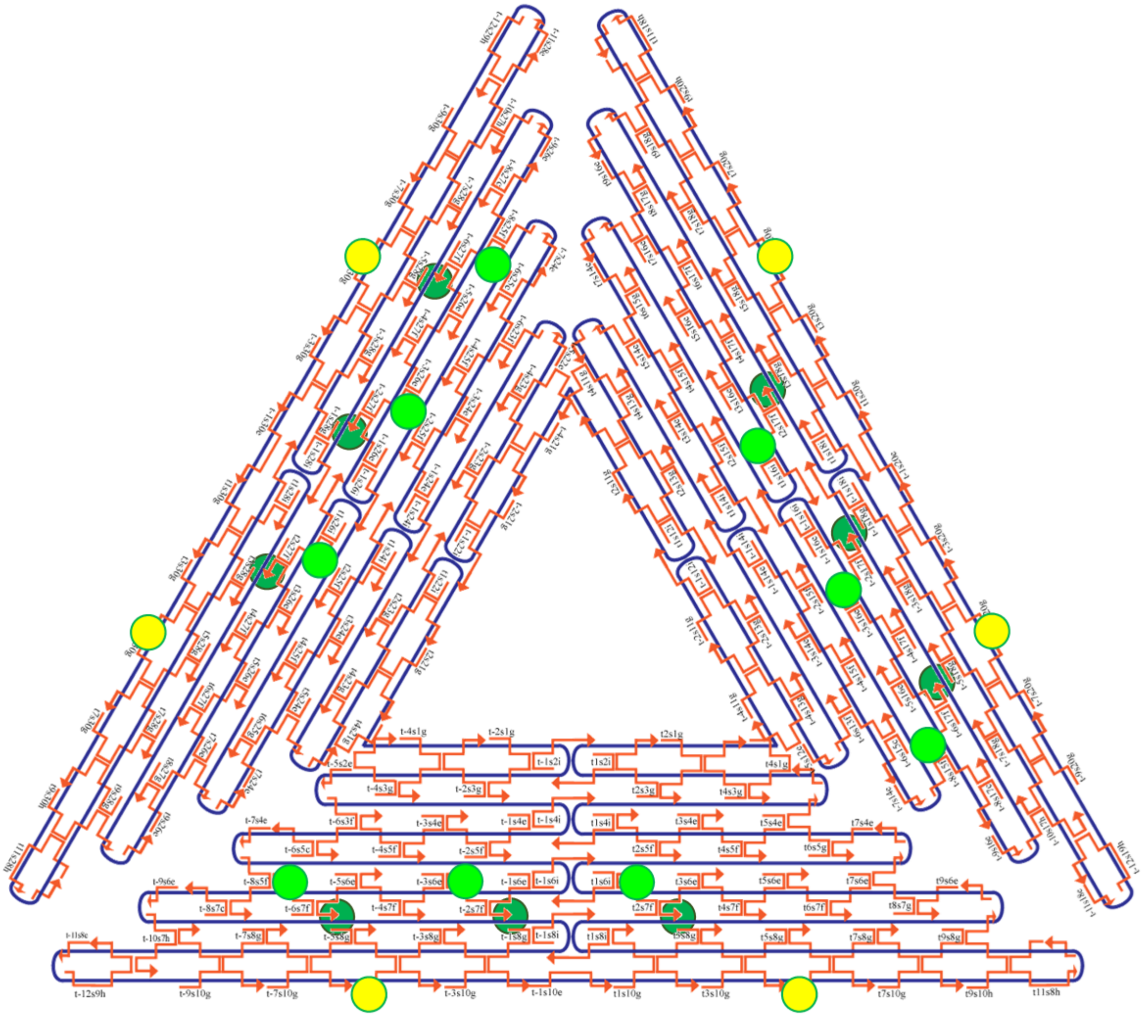

Figure S2. Scaffold routing and staple pattern of V9/9-DON. Vancomycin sites are indicated in green with light and dark green corresponding to opposite sides. Biotin sites are indicated in yellow.

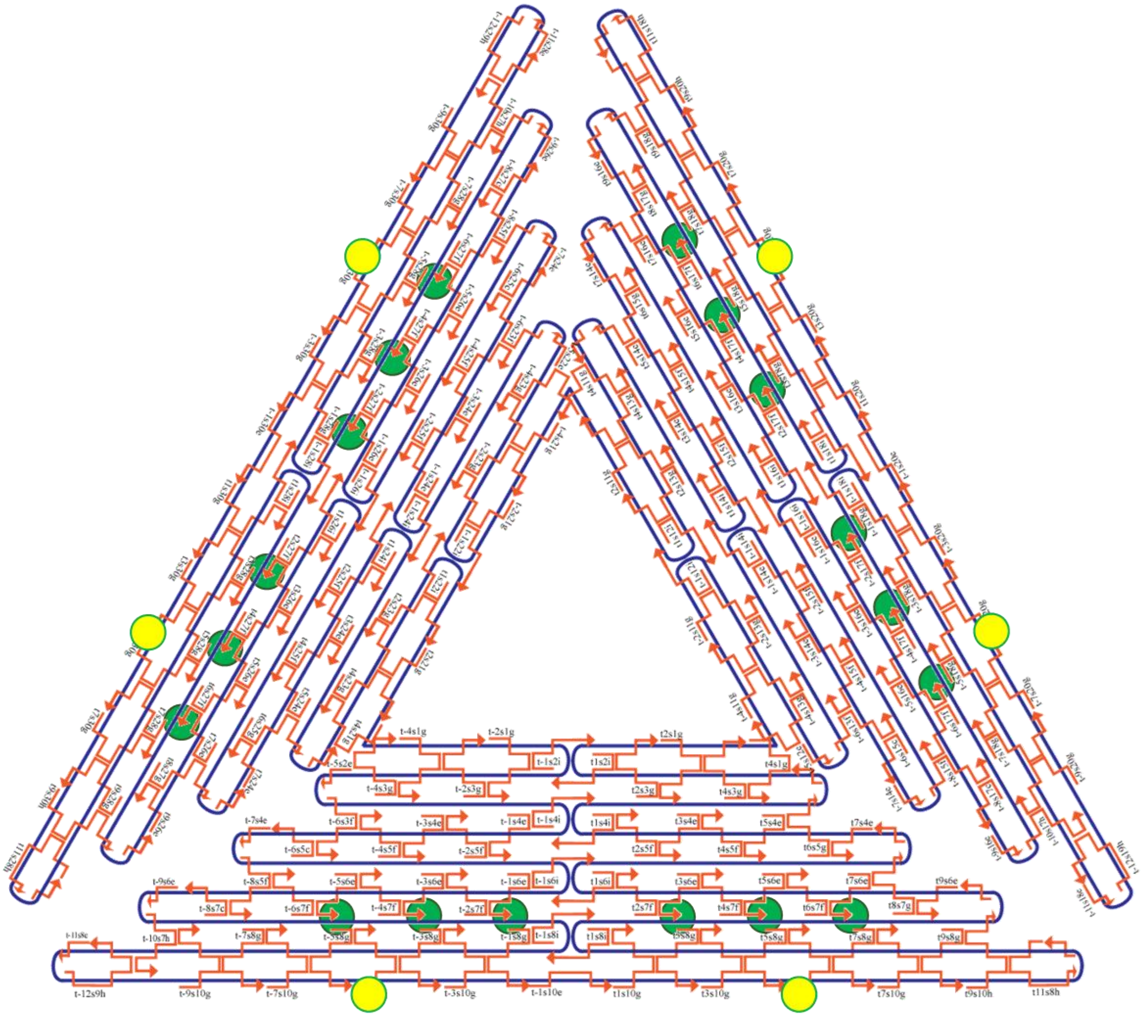

Figure S3. Scaffold routing and staple pattern of V18-DON. Vancomycin sites are indicated in green. Biotin sites are indicated in yellow.

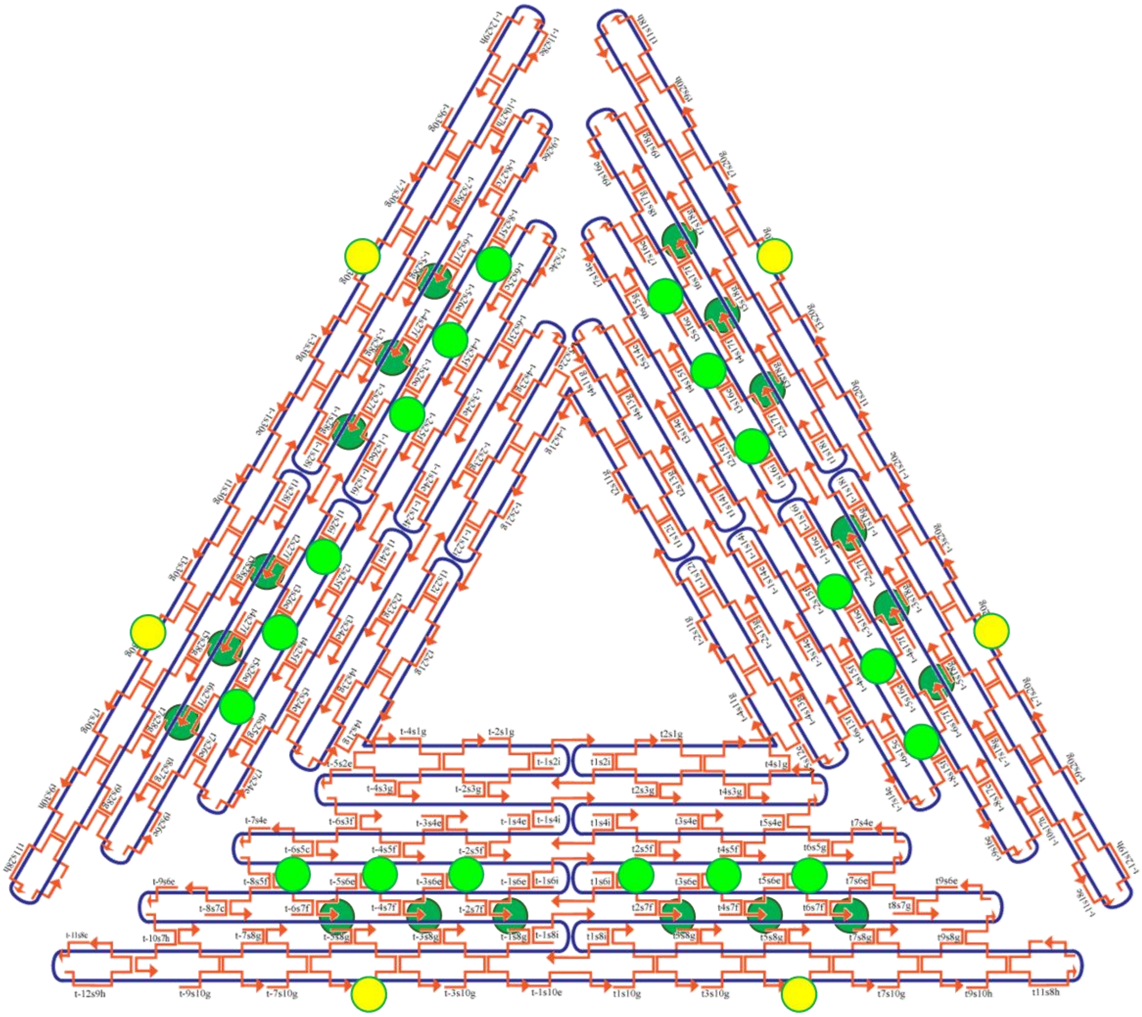

Figure S4. Scaffold routing and staple pattern of V18/18-DON. Vancomycin sites are indicated in green with light and dark green corresponding to opposite sides. Biotin sites are indicated in yellow.

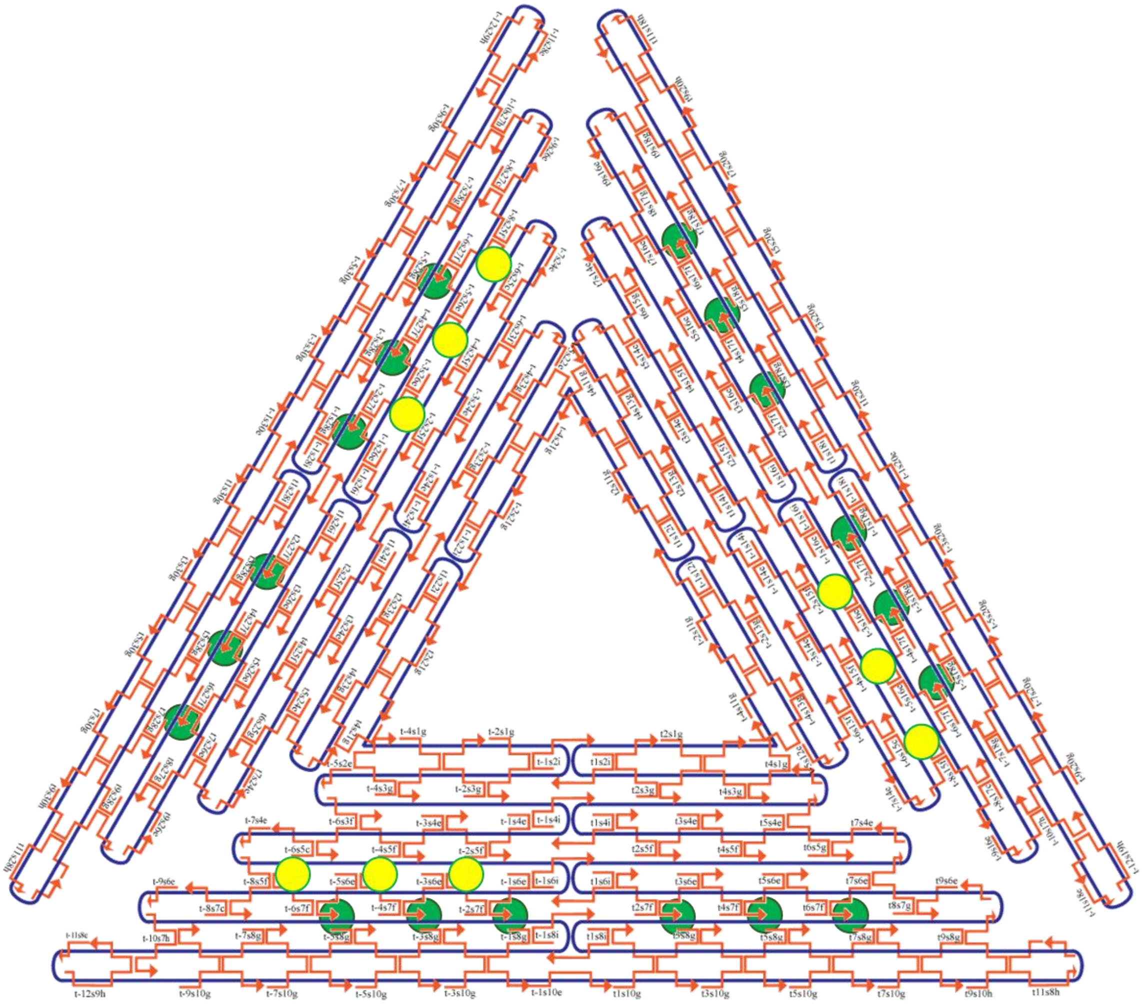

Figure S5. Scaffold routing and staple pattern of V18/B9-DON. Vancomycin sites are indicated in green. Biotin sites are indicated in yellow.

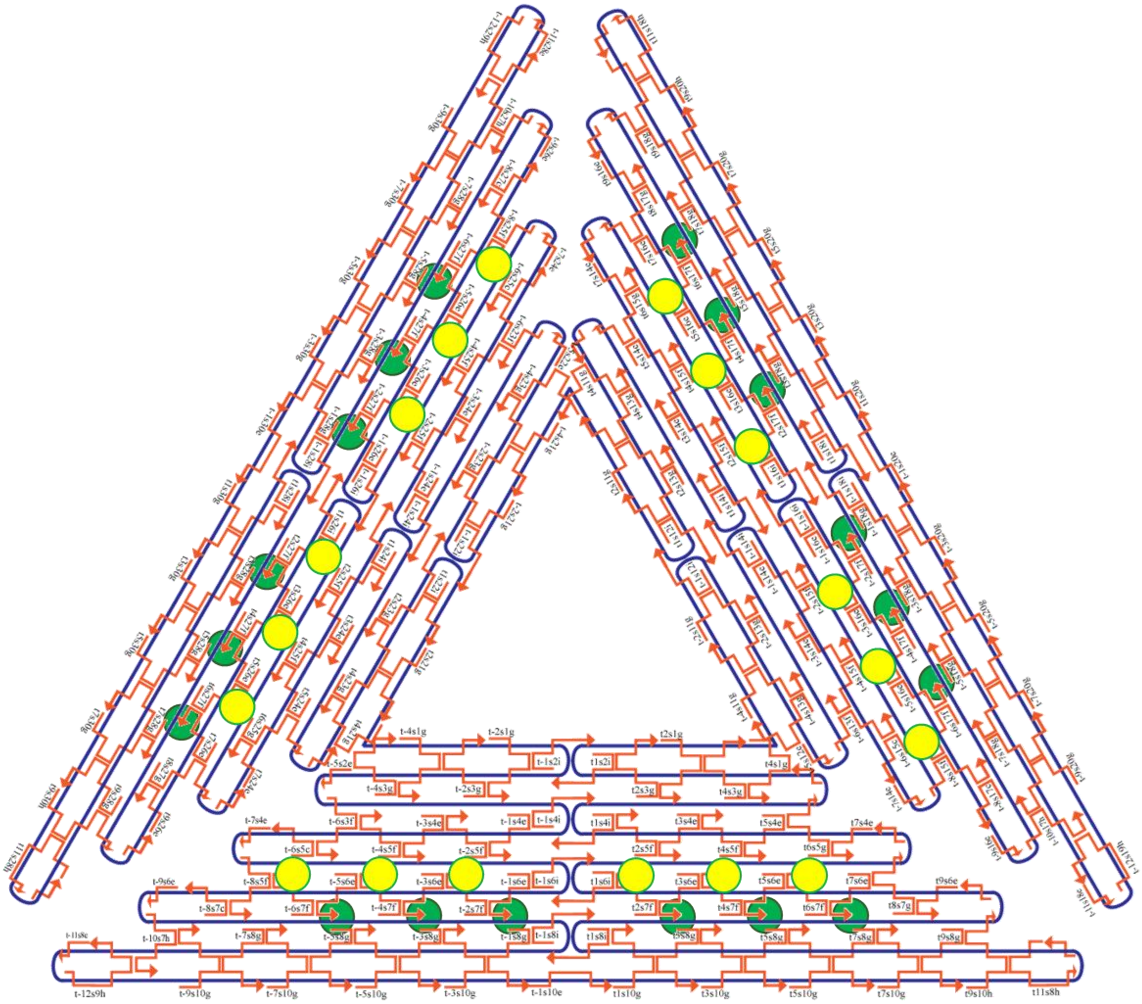

Figure S6. Scaffold routing and staple pattern of V18/B18-DON. Vancomycin sites are indicated in green. Biotin sites are indicated in yellow.

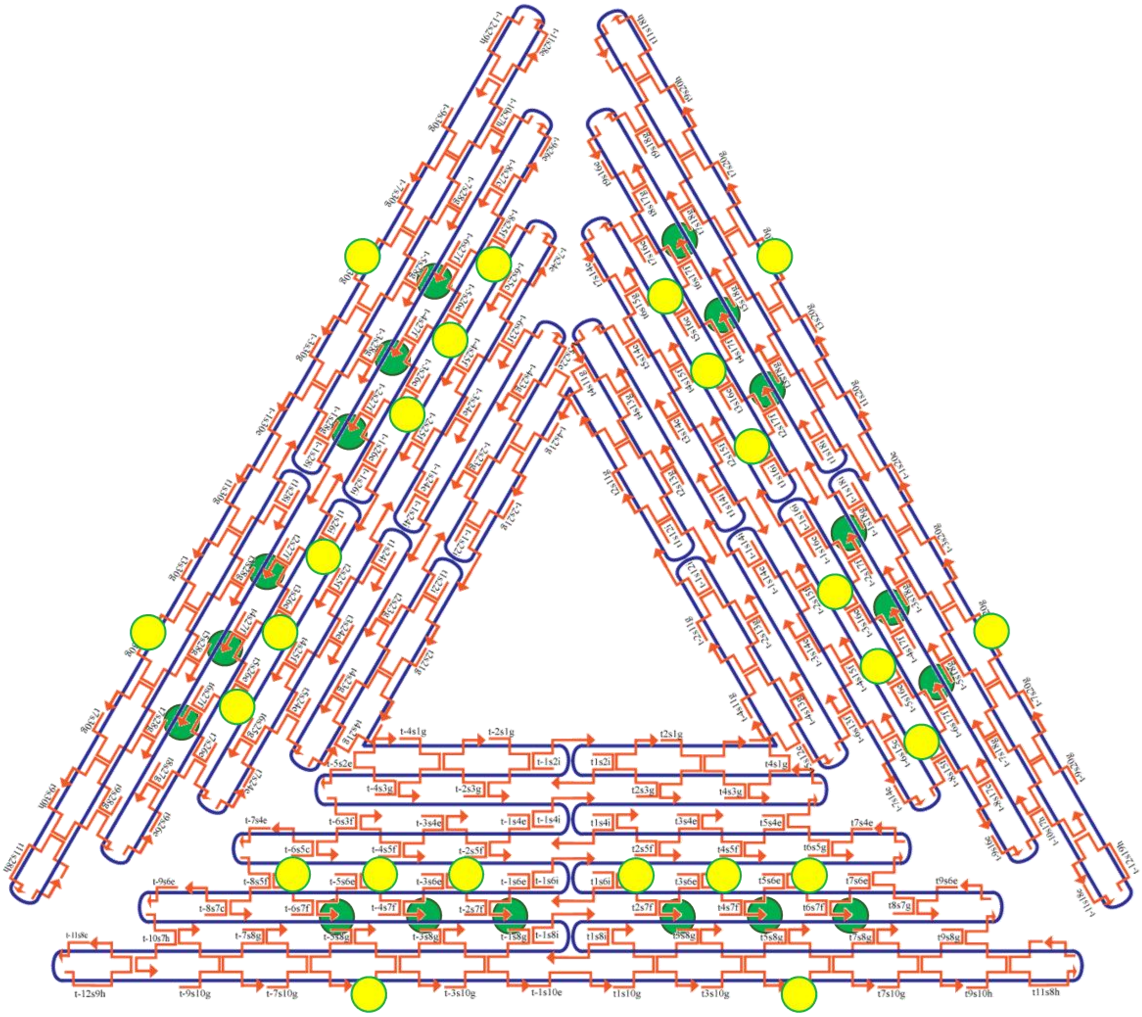

Figure S7. Scaffold routing and staple pattern of V18/B24-DON. Vancomycin sites are indicated in green. Biotin sites are indicated in yellow.

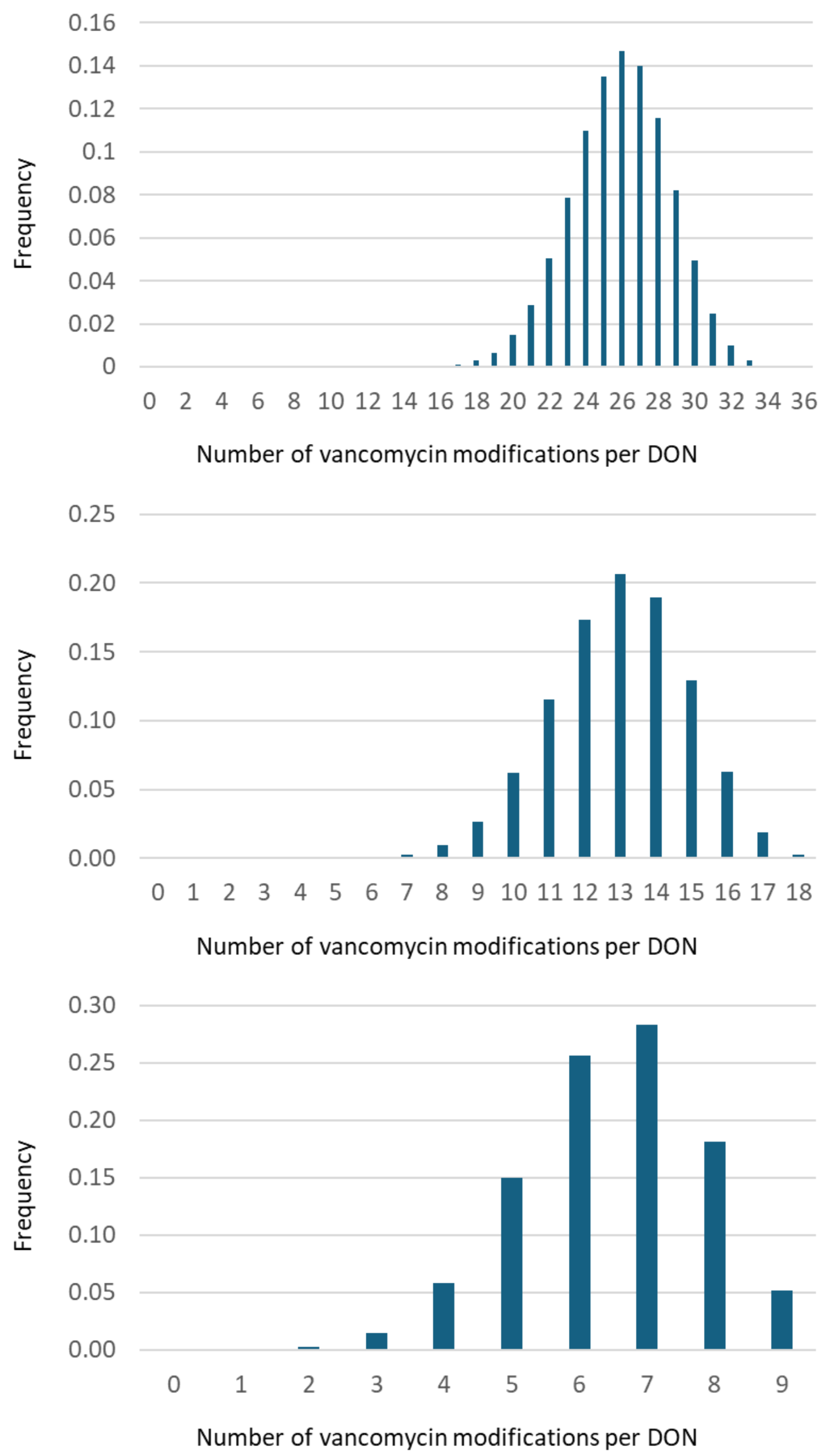

Figure S8. Computational simulations[1] of the distributions of vancomycin modifications presented on DONs with a maximum of 36 (top), 18 (center), and 9 (bottom) modifications. An average staple conjugation yield of 85 % and an average staple incorporation yield of 85 % was used as estimated previously.[1]

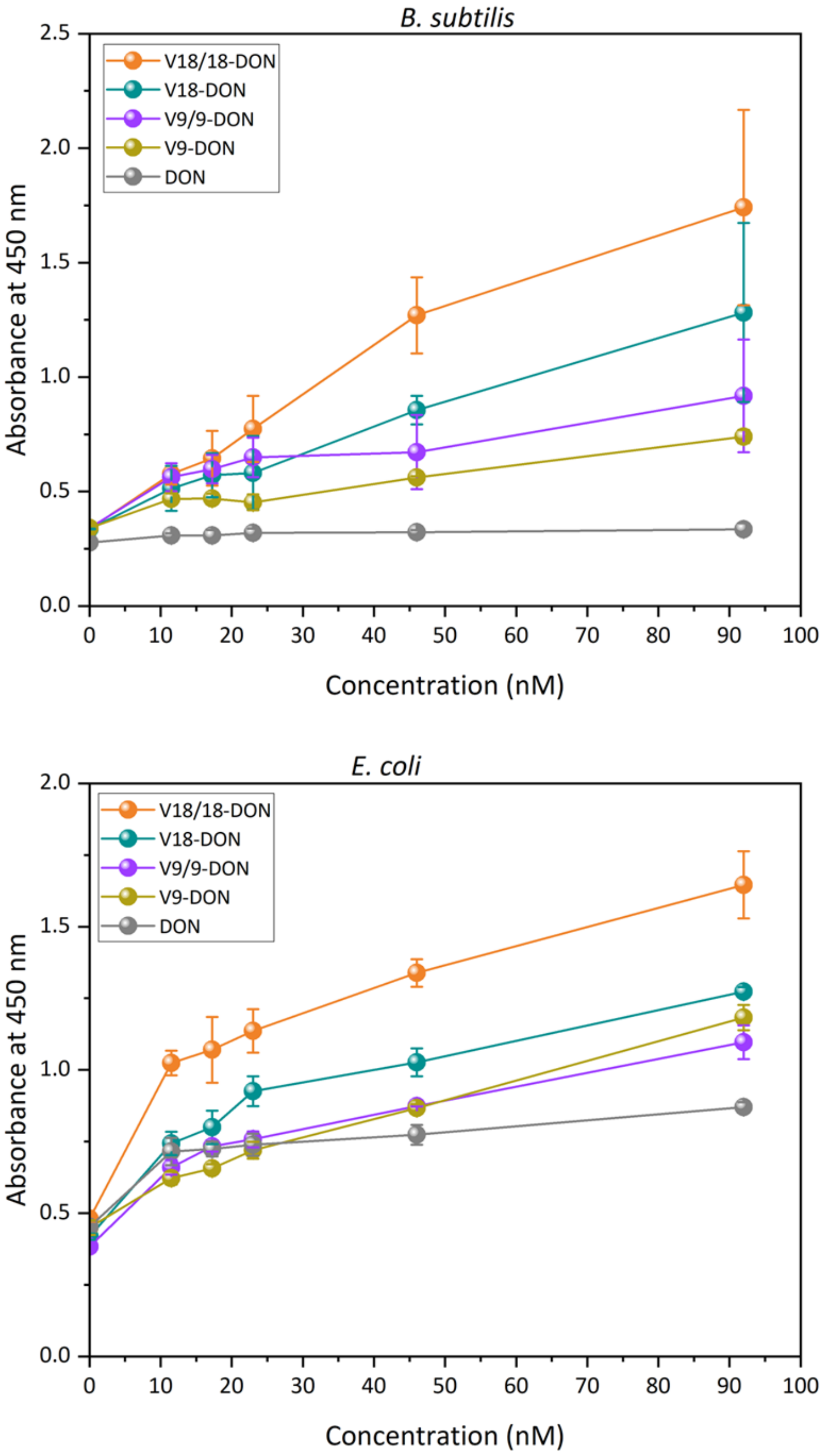

Figure S9. Non-normalized absorbance values for the different DON variants bound to *B. subtilis* and *E. coli*.

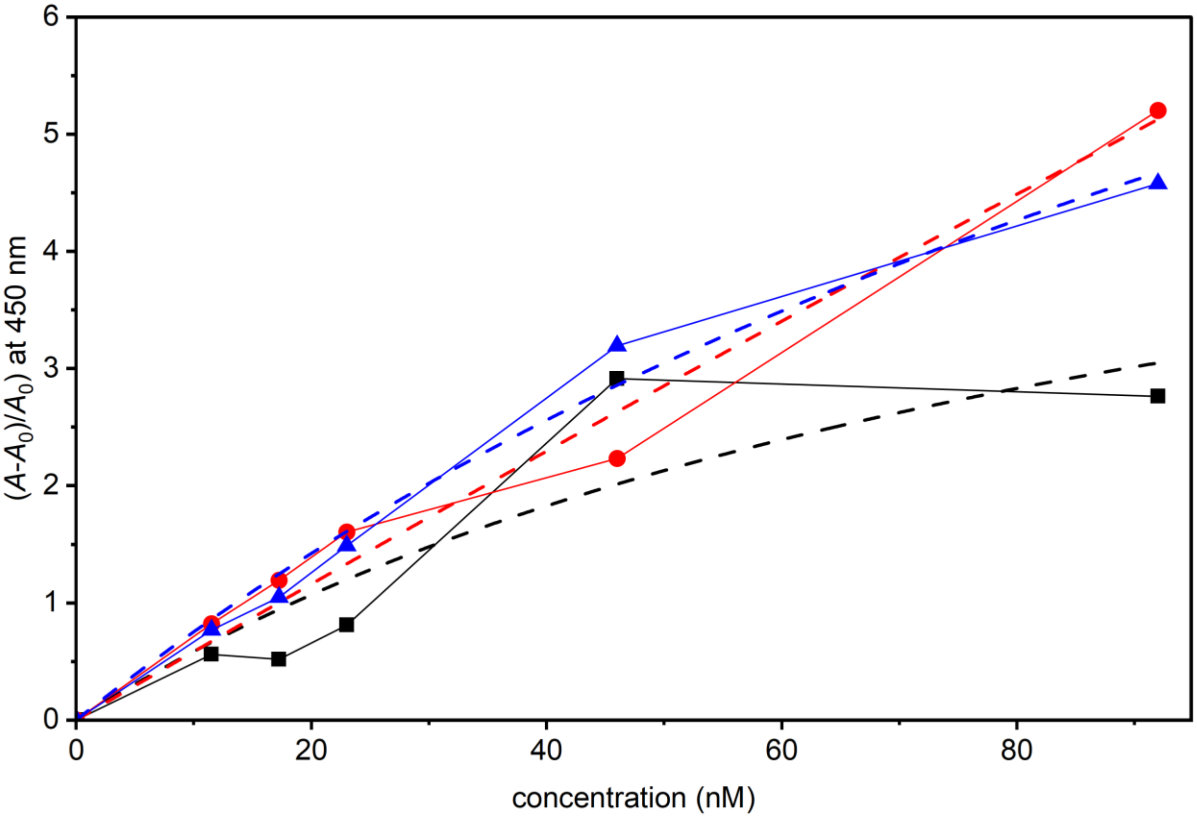

Figure S10. Normalized absorbance values for V18/18-DON bound to *B. subtilis* and fits (dashed lines) for determining EC_1.

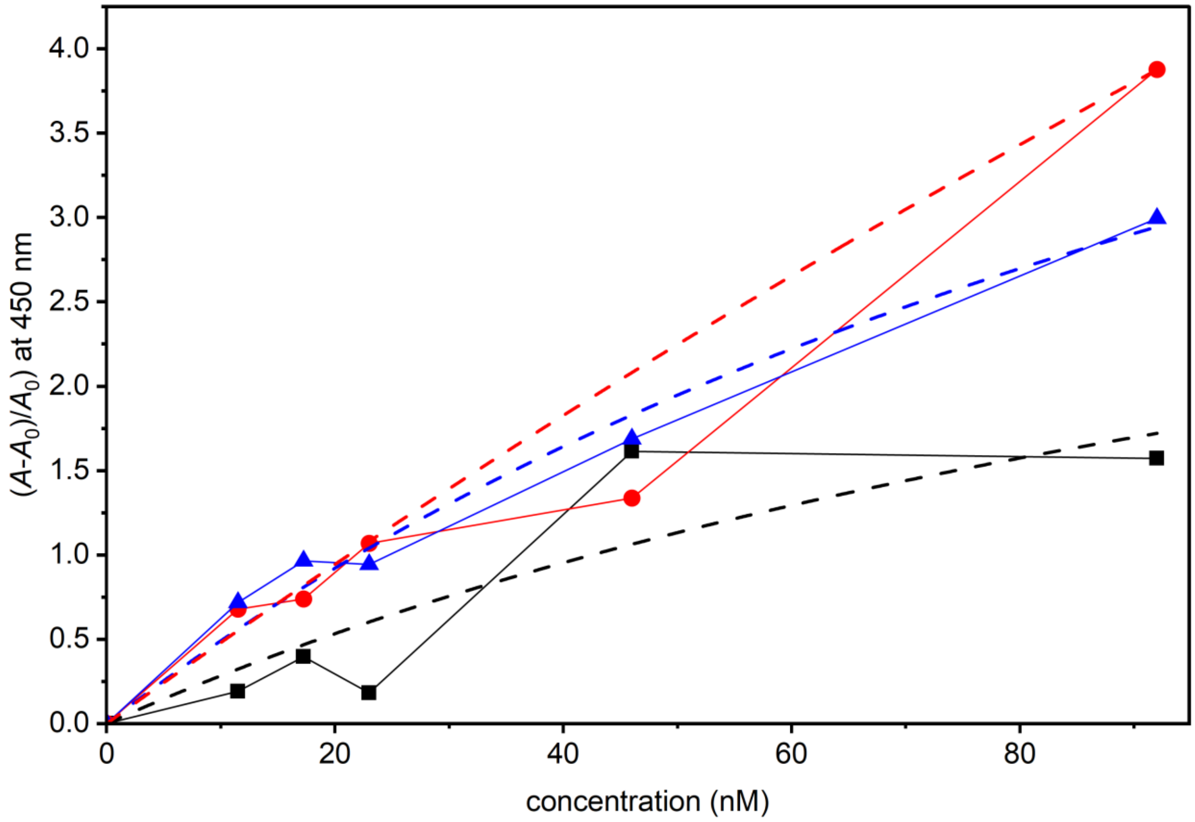

Figure S11. Normalized absorbance values for V18-DON bound to *B. subtilis* and fits (dashed lines) for determining EC_1.

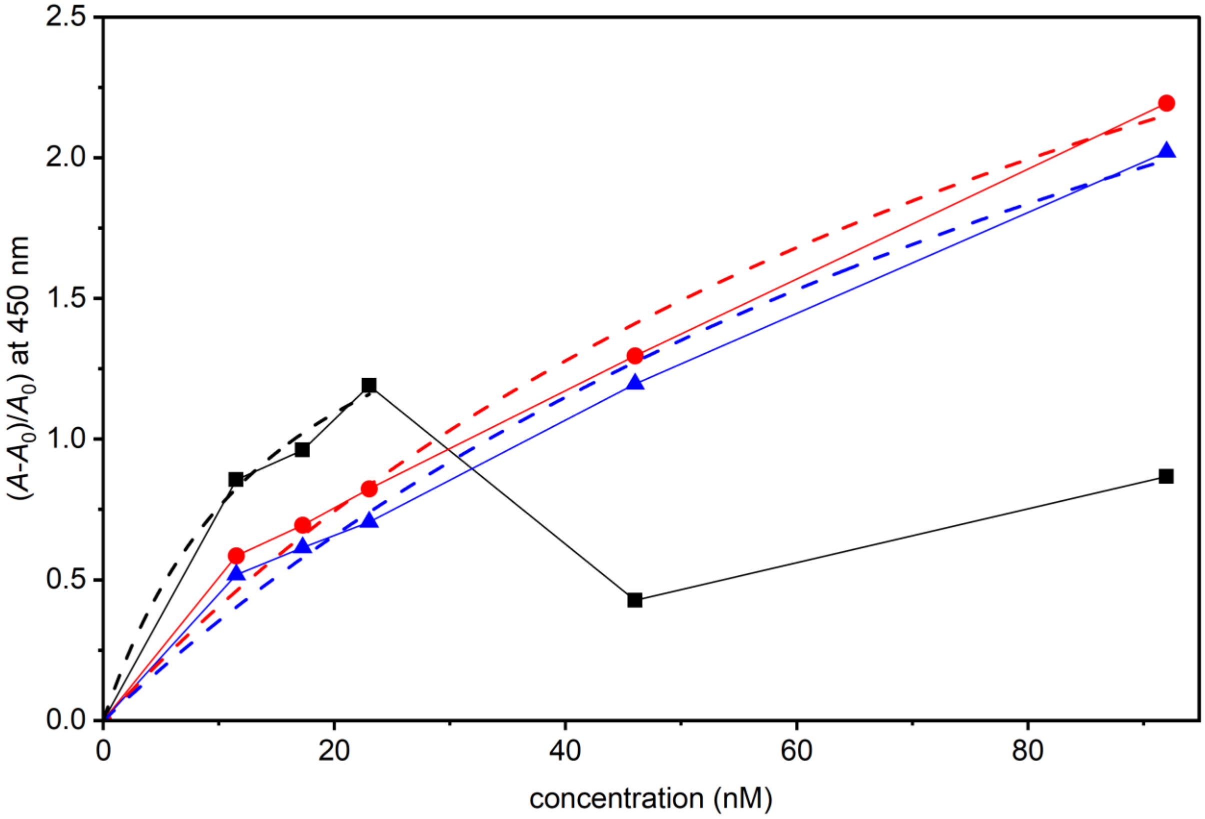

Figure S12. Normalized absorbance values for V9/9-DON bound to *B. subtilis* and fits (dashed lines) for determining EC_1. Note that the black dataset was fit only until 23 nM due to the observed drop in signal for higher concentrations.

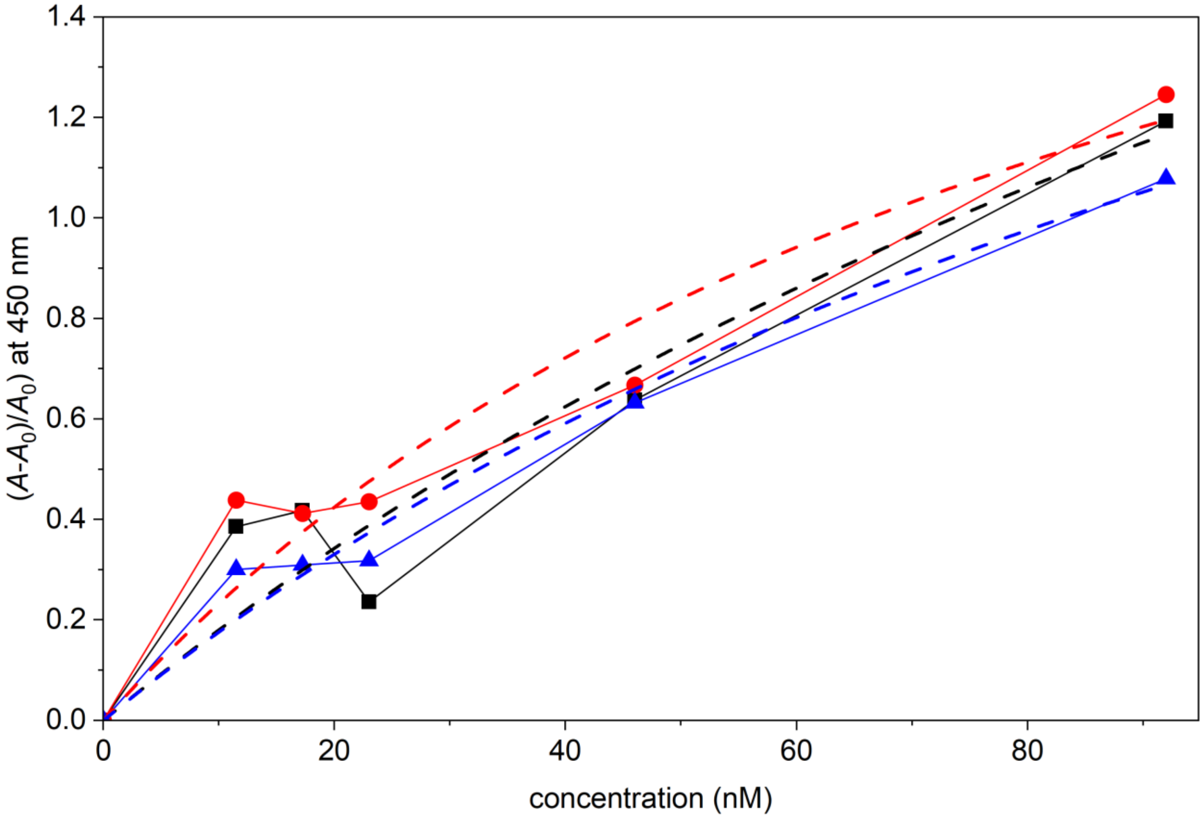

Figure S13. Normalized absorbance values for V9-DON bound to *B. subtilis* and fits (dashed lines) for determining EC_1.

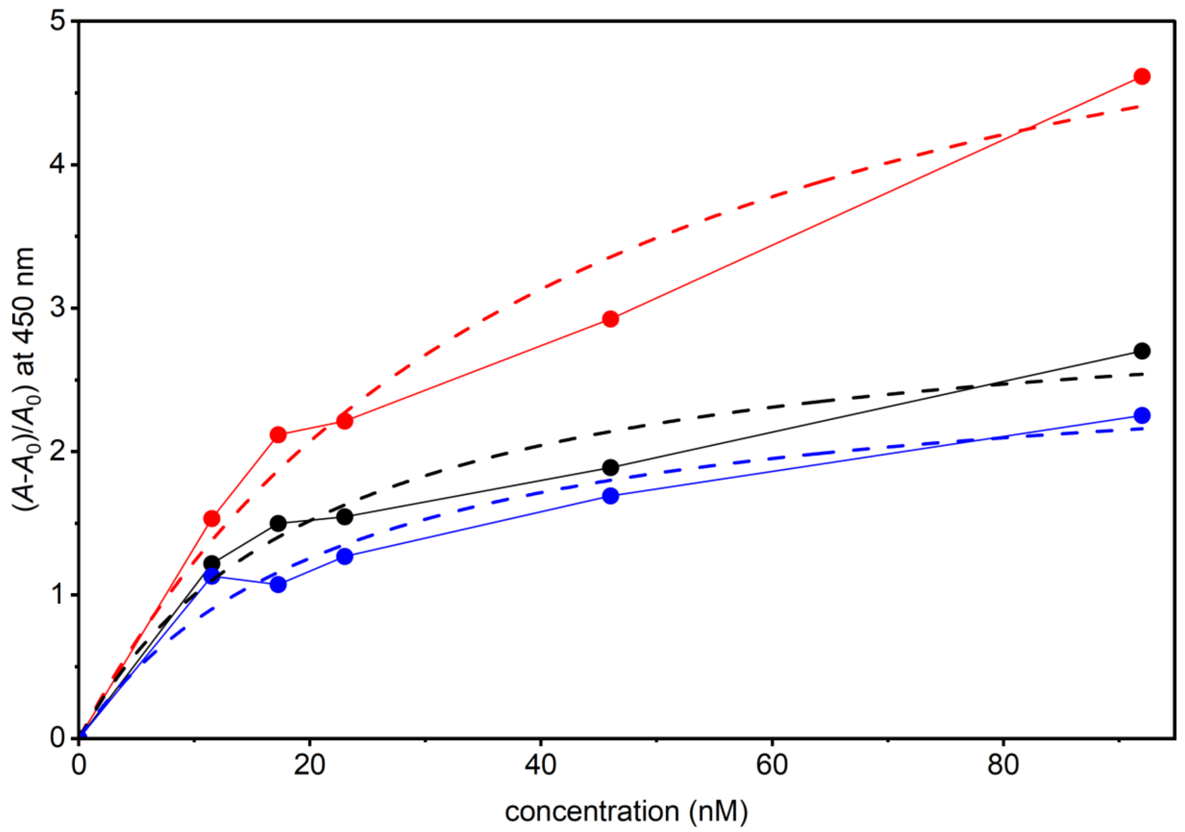

Figure S14. Normalized absorbance values for V18/18-DON bound to *E. coli* and fits (dashed lines) for determining EC_1.

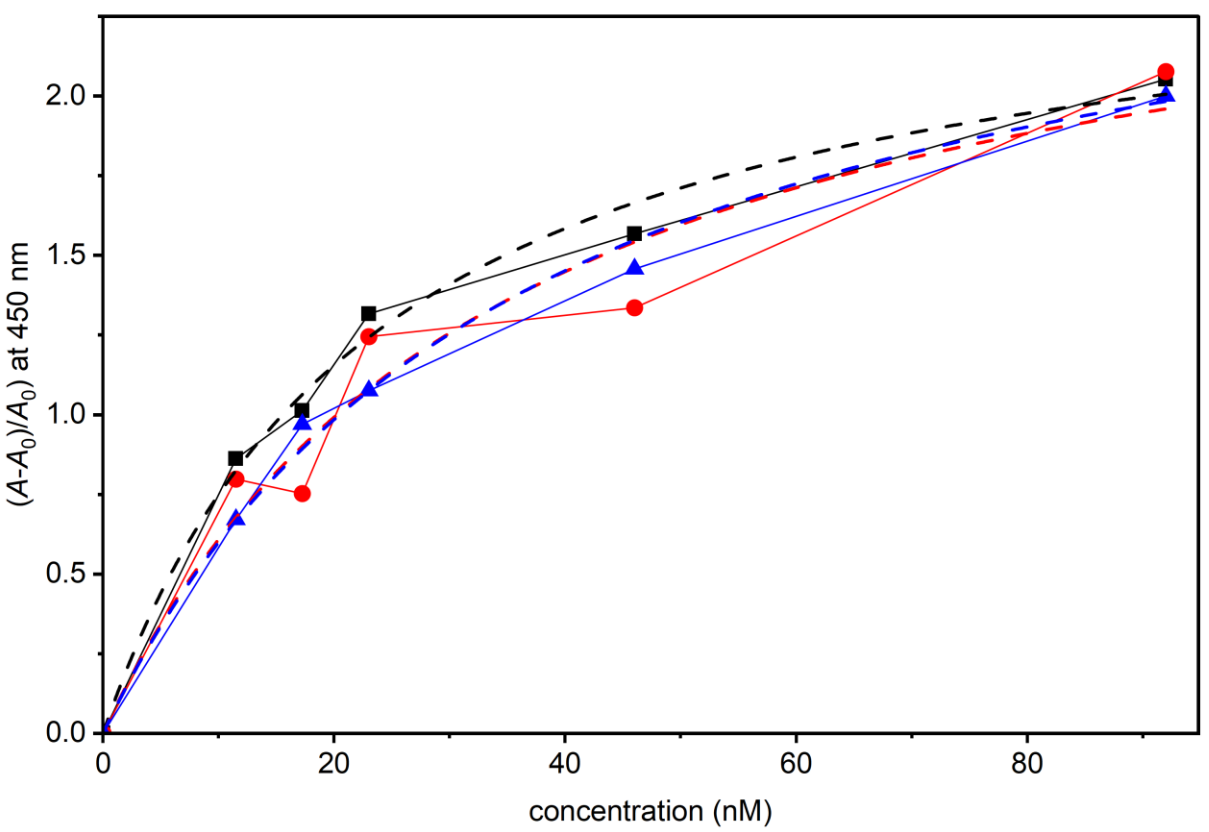

Figure S15. Normalized absorbance values for V18-DON bound to *E. coli* and fits (dashed lines) for determining EC_1.

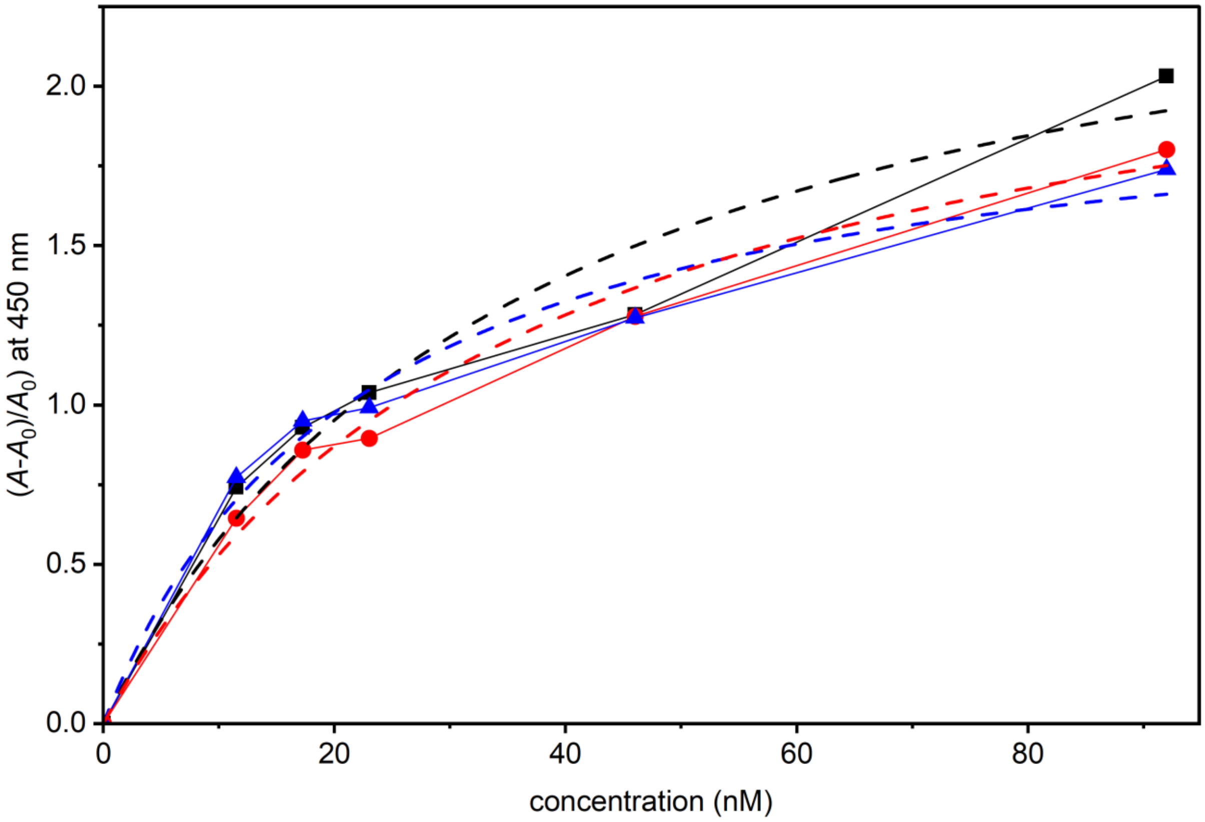

Figure S16. Normalized absorbance values for V9/9-DON bound to *E. coli* and fits (dashed lines) for determining EC_1.

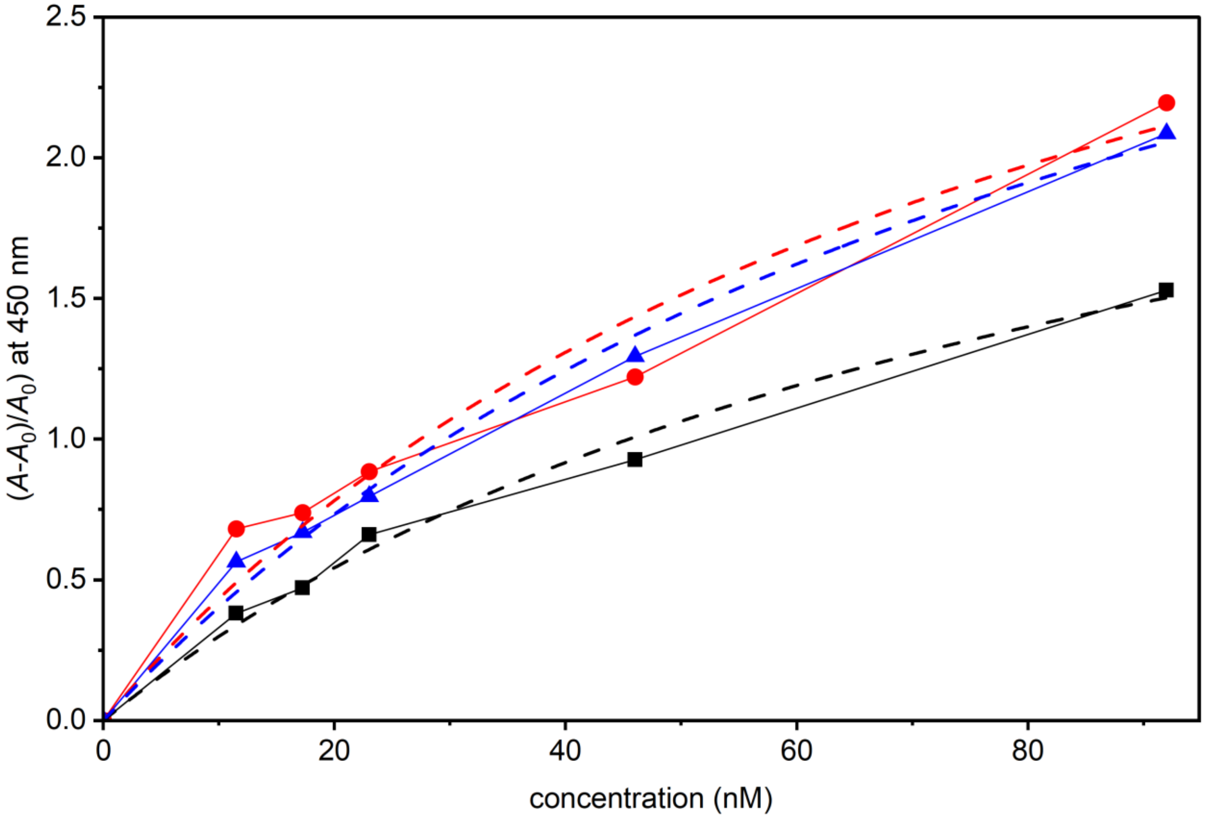

Figure S17. Normalized absorbance values for V9-DON bound to *E. coli* and fits (dashed lines) for determining EC_1.

Table S1. Amino-modified staples used for vancomycin conjugation. Staple IDs follow the original nomenclature introduced by Rothemund.[2] Poly-T spacers are shown in bold.

ID	Sequence 5' → 3'
t2s7f	AAA GAC AAC ATT TTC GGT CAT AGC CAA AAT CA**T TTT TT**-C7-Amino
t2s17f	AAC CAG ACG TTT AGC TAT ATT TTC TTC TAC TA**T TTT TT**-C7-Amino
t2s27f	GGA CAT TCA CCT CAA ATA TCA AAC ACA GTT GA**T TTT TT**-C7-Amino
t3s6e	CAC CGG AAA GCG CGT TTT CAT CGG AAG GGC GA**T TTT TT**-C7-Amino
t3s16e	CAT CCA ATA AAT GGT CAA TAA CCT CGG AAG CA**T TTT TT**-C7-Amino
t3s26e	TTA TCT AAA GCA TCA CCT TGC TGA TGG CCA AC**T TTT TT**-C7-Amino
t4s7f	GGA GGG AAT TTA GCG TCA GAC TGT CCG CCT CC**T TTT TT**-C7-Amino
t4s17f	GAT TAG AGA TTA GAT ACA TTT CGC AAA TCA TA**T TTT TT**-C7-Amino
t4s27f	TGA CCT GAC AAA TGA AAA ATC TAA AAT ATC TT**T TTT TT**-C7-Amino
t5s6e	TCA GAA CCC AGA ATC AAG TTT GCC GGT AAA TA**T TTT TT**-C7-Amino
t5s16e	TTA GCA AAT AGA TTT AGT TTG ACC AGT ACC TT**T TTT TT**-C7-Amino
t5s26e	TAA TAG ATC GCT GAG AGC CAG CAG AAG CGT AA**T TTT TT**-C7-Amino
t6s7f	ATT AAA GGC CGT AAT CAG TAG CGA GCC ACC CT**T TTT TT**-C7-Amino
t6s17f	TAA GAG GTC AAT TCT GCG AAC GAG ATT AAG CA**T TTT TT**-C7-Amino
t6s27f	CAA TAT TTG CCT GCA ACA GTG CCA TAG AGC CG**T TTT TT**-C7-Amino
t7s6e	AGA GCC GCA CCA TCG ATA GCA GCA TGA ATT AT**T TTT TT**-C7-Amino
t7s16e	TAA AGC TAT ATA ACA GTT GAT TCC CAT TTT TG**T TTT TT**-C7-Amino
t7s26e	TTG AGG ATG GTC AGT ATT AAC ACC TTG AAT GG**T TTT TT**-C7-Amino
t-1s6e	TTA GTA TCG CCA ACG CTC AAC AGT CGG CTG TC**T TTT TT**-C7-Amino
t-1s16e	ATT CGG TCT GCG GGA TCG TCA CCC GAA ATC CG**T TTT TT**-C7-Amino
t-1s26e	GCC AGT GCG ATC CCC GGG TAC CGA GTT TTT CT**T TTT TT**-C7-Amino
t-2s7f	TCA ATA ATA GGG CTT AAT TGA GAA TCA TAA TT**T TTT TT**-C7-Amino
t-2s17f	ATT GTG TCT CAG CAG CGA AAG ACA CCA TCG CC**T TTT TT**-C7-Amino
t-2s27f	CCA GGG TGG CTC GAA TTC GTA ATC CAG TCA CG**T TTT TT**-C7-Amino
t-3s6e	CAC CGG AAT CGC CAT ATT TAA CAA AAT TTA CG**T TTT TT**-C7-Amino
t-3s16e	GAC AAC AAG CAT CGG AAC GAG GGT GAG ATT TG**T TTT TT**-C7-Amino
t-3s26e	GGT TTT CCA TGG TCA TAG CTG TTT GAG AGG CG**T TTT TT**-C7-Amino
t-4s7f	CCC ATC CTC GCC AAC ATG TAA TTT AAT AAG GC**T TTT TT**-C7-Amino
t-4s17f	GTA CAA CGA GCA ACG GCT ACA GAG GAT ACC GA**T TTT TT**-C7-Amino
t-4s27f	CGC GCG GGC CTG TGT GAA ATT GTT GGC GAT TA**T TTT TT**-C7-Amino
t-5s6e	GTG TGA TAA GGC AGA GGC ATT TTC AGT CCT GA**T TTT TT**-C7-Amino
t-5s16e	AAC AGC TTG CTT TGA GGA CTA AAG CGA TTA TA**T TTT TT**-C7-Amino
t-5s26e	TGC TGC AAA TCC GCT CAC AAT TCC CAG CTG CA**T TTT TT**-C7-Amino
t-6s7f	AAT AGA TAG AGC CAG TAA TAA GAG ATT TAA TG**T TTT TT**-C7-Amino
t-6s17f	ACC CCC AGA CTT TTT CAT GAG GAA CTT GCT TT**T TTT TT**-C7-Amino
t-6s27f	TGT CGT GCA CAC AAC ATA CGA GCC ACG CCA GC**T TTT TT**-C7-Amino

Table S2. Biotin-modified edge staples for ELONA measurements. Staple IDs follow the original nomenclature introduced by Rothemund.[2] Poly-T spacers are shown in bold.

**ID**	**Sequence 5' → 3'**
t-5s10g	Biotin-**TTT TTT TTT TTT** TCC CAA TCC AAA TAA GAT TAC CGC GCC CAA TAA ATA ATA T
t-5s20g	Biotin-**TTT TTT TTT TTT** ACC AGT CAG GAC GTT GGA ACG GTG TAC AGA CCG AAA CAA A
t-5s30g	Biotin-**TTT TTT TTT TTT** CTA AAT CGG AAC CCT AAG CAG GCG AAA ATC CTT CGG CCA A
t5s10g	Biotin-**TTT TTT TTT TTT** GAT AAC CCA CAA GAA TGT TAG CAA ACG TAG AAA ATT ATT C
t5s20g	Biotin-**TTT TTT TTT TTT** AAC ACT ATC ATA ACC CAT CAA AAA TCA GGT CTC CTT TTG A
t5s30g	Biotin-**TTT TTT TTT TTT** TTA AAG GGA TTT TAG ATA CCG CCA GCC ATT GCG GCA CAG A

Table S3. Biotin-modified surface staples for ELONA measurements used in V18/B9-DON, V18/B18-DON, and V18/B24-DON. Staple IDs follow the original nomenclature introduced by Rothemund.[2] Poly-T spacers are shown in bold.

**ID**	**Sequence 5' → 3'**
t2s7f	AAA GAC AAC ATT TTC GGT CAT AGC CAA AAT CA**T TTT TTT T**-Biotin
t2s17f	AAC CAG ACG TTT AGC TAT ATT TTC TTC TAC TA**T TTT TTT T**-Biotin
t2s27f	GGA CAT TCA CCT CAA ATA TCA AAC ACA GTT GA**T TTT TTT T**-Biotin
t4s7f	GGA GGG AAT TTA GCG TCA GAC TGT CCG CCT CC**T TTT TTT T**-Biotin
t4s17f	GAT TAG AGA TTA GAT ACA TTT CGC AAA TCA TA**T TTT TTT T**-Biotin
t4s27f	TGA CCT GAC AAA TGA AAA ATC TAA AAT ATC TT**T TTT TTT T**-Biotin
t6s7f	ATT AAA GGC CGT AAT CAG TAG CGA GCC ACC CT**T TTT TTT T**-Biotin
t6s17f	TAA GAG GTC AAT TCT GCG AAC GAG ATT AAG CA**T TTT TTT T**-Biotin
6s27f	CAA TAT TTG CCT GCA ACA GTG CCA TAG AGC CG**T TTT TTT T**-Biotin
t-2s7f	TCA ATA ATA GGG CTT AAT TGA GAA TCA TAA TT**T TTT TTT T**-Biotin
t-2s17f	ATT GTG TCT CAG CAG CGA AAG ACA CCA TCG CC**T TTT TTT T**-Biotin
t-2s27f	CCA GGG TGG CTC GAA TTC GTA ATC CAG TCA CG**T TTT TTT T**-Biotin
t-4s7f	CCC ATC CTC GCC AAC ATG TAA TTT AAT AAG GC**T TTT TTT T**-Biotin
t-4s17f	GTA CAA CGA GCA ACG GCT ACA GAG GAT ACC GA**T TTT TTT T**-Biotin
t-4s27f	CGC GCG GGC CTG TGT GAA ATT GTT GGC GAT TA**T TTT TTT T**-Biotin
t-6s7f	AAT AGA TAG AGC CAG TAA TAA GAG ATT TAA TG**T TTT TTT T**-Biotin
t-6s17f	ACC CCC AGA CTT TTT CAT GAG GAA CTT GCT TT**T TTT TTT T**-Biotin
t-6s27f	TGT CGT GCA CAC AAC ATA CGA GCC ACG CCA GC**T TTT TTT T**-Biotin

References

[1] Ö. Coşkuner Leineweber, B. K. Pothineni, N. Schumann, U. Hofmann, C. Möser, D. M. Smith, G. Grundmeier, Y. Zhang, A. Keller, *Small Struct.* **2025**, *6*, e202500246.

[2] P. W. K. Rothemund, *Nature* **2006**, *440*, 297–302.
